# Microbe- plant interaction as a sustainable tool for mopping up heavy metal contaminated sites

**DOI:** 10.1186/s12866-022-02587-x

**Published:** 2022-07-07

**Authors:** Ahmed A. Sorour, Heba Khairy, Eman H. Zaghloul, Heba A. H. Zaghloul

**Affiliations:** 1 Department of Botany and Microbiology, Faculty of Science, Alexandria University, Moharam Bek, Alexandria, 21511 Egypt; 2 National Institute of Oceanography and Fisheries, NIOF, Cairo, Egypt

**Keywords:** Plant Growth Promoting Rhizobacteria (PGPR), Metal accumulation, Metal tolerance, *Helianthus annuus*, Heavy Metal ATPase, *HMA3*, *HMA4*

## Abstract

**Background:**

Phytoremediation is a green technology that removes heavy metal (HM) contamination from the environment by using HM plant accumulators. Among soil microbiota, plant growth promoting bacteria (PGPR) have a role influencing the metal availability and uptake.

**Methods:**

This current study evaluates the plant growth promoting qualities of microbial flora isolated from rhizosphere, plant roots, and marine aquatic HMs polluted environments in Alexandria through several biochemical and molecular traits. Metal contents in both collected soils and plant tissues were measured. Transcript levels of marker genes (*HMA3* and *HMA4*) were analyzed.

**Results:**

Three terrestrial and one aquatic site were included in this study based on the ICP-MS identification of four HMs (Zn, Cd, Cu, and Ni) or earlier reports of HMs contamination. Using the VITEK2 bacterial identification system, twenty-two bacteria isolated from these loci were biochemically described. *Pseudomonas* and *Bacillus* were the most dominant species. Furthermore, the soil microbiota collected from the most contaminated HMs site with these two were able to enhance the *Helianthus annuus* L. hyper-accumulation capacity significantly. Specifically, sunflower plants cultivated in soils with HMs adapted bacteria were able to accumulate about 1.7–2.5-folds more Zn and Cd in their shoots, respectively.

**Conclusion:**

The influence of PGPR to stimulate crop growth under stress is considered an effective strategy. Overall, our findings showed that plants cultivated in HMs contaminated sites in the presence of PGPR were able to accumulate significant amounts of HMs in several plant parts than those cultivated in soils lacking microbiota.

**Supplementary Information:**

The online version contains supplementary material available at 10.1186/s12866-022-02587-x.

## Background

Heavy metals (HMs) are toxic and non-biodegradable pollutants. For natural and anthropogenic factors, these metals may reach high concentrations in some environments. Phytoremediation is one of the most promising strategies to restore and remediate these loci in an ecologically and environmentally sound and safe way [1]. All plants possess a certain level of basal tolerance to heavy metals in order to survive in soils, which are characterized by a pronouncedly heterogeneous composition, and fluctuating bioavailability of metals [2]. Both metal hyperaccumulators and all plants that grow on metalliferous soils possess a clearly higher level of tolerance named “hypertolerance” [3, 4]. The most widespread mechanism of plant metal tolerance, common to non-adapted plants and found in more than 99% of the species on metalliferous soils, is to avoid either the uptake from soil or root-to-shoot translocation of metals, termed “exclusion” strategy based on assessment of leaf metal concentrations in the field [5]. Furthermore, acquisition and sequestration are another strategies for heavy metal detoxification and tolerance [6]. The alternative strategy, metal hyperaccumulation, is always associated with hypertolerance, and it highlights the importance of other mechanisms of internal metal detoxification involving cytoplasmic metal chelation, efflux from sensitive cells and sequestration in vacuoles and cell walls of cells suited for metal storage in non-toxic form. Phytoremediation is the green technique for the removal of HMs from contaminated sites [7]. Phytoextraction is the use of metabolic potentials of (hyper)accumulator plants to extract metal contaminants from soils which can improve their fertility. *Helianthus annus* has the capacity to accumulate HMs and considered a suitable crop plant for mopping up contaminated sites [8, 9]. Heavy metal ATPases such as *HMA3* and *HMA4* are responsible for different accumulation and tolerance capacities of Zn and Cd. Overexpression of *Noccaea caerulescens Heavy Metal ATPase 3* (*TcHMA3*) enhanced the tolerance to Zn and Cd. *NcHMA3* encodes a tonoplast transport protein specific for cadmium transport, which is responsible for sequestration of Cd into the leaf vacuoles [10]. *HMA4* (*Heavy Metal ATPase 4*) is a gene that encode a plasma membrane protein transition metals transporter. The encoded proteins enhance the partitioning of metals from the root into the shoot [11, 12].

HMs pollution of the soil is not the only stressor to which the plant is exposed. High salinity levels are another major stressor. Because of the increased contamination of salinized fields with HMs, plant adaptation to these dual pollutants is becoming a more serious problem. In the meantime, around 25% of all land is salty to some extent [13]. Salinization is a drawback effect of climate change and desertification process [14]. Osmotic stress caused by salinity reduces the availability of critical elements including, Ca, K, Zn and Fe, resulting in nutrient deficiency in plants [15]. Moreover, the interaction of HM ions with chloride in soil is thought to be an important factor in determining the selectivity of HM uptake by plants [13]. Similarly, plants mitigate the drastic effects of salinity by several strategies. For example, hormone stimulation, ion exchange, antioxidant enzymes, and signaling cascade activation [16].

Aside from plant mechanisms for dealing with HMs and salinity, Plant Growth Promoting Rhizobacteria (PGPR) play an important role in boosting the plant capacity to withstand these stresses. The PGPR include several species from genus *Bacillus*, *Pseudomonas*, *Azotobacter*, *Azospirillum*, *Arthrobacter*, *Achromobacter*, *Enterobacter*, *Streptomyces*, etc. These PGPR are known to develop several mechanisms to alleviate the metal toxicity like metal-detoxification, biosorption, bioaccumulation, bioleaching, bioexclusion, metal-solubilization, acidification, protonation, chelation and metal-immobilization [17]. Furthermore, these PGPR along with other microbes assist the plant growth by other mechanisms including, production of siderophores [18], indole-3-acetic acid (IAA) and HCN gas [19], along with enhancing the mineral nutrients bioavailability like phosphorous [20]. Moreover, PGPR suppress plant pathogenic invaders. Overall, the plant and PGPR systems work together to maintain plant development in the face of biotic and abiotic stress.

The aim of the current study was to evaluate a set of plant growth promoting traits of microbial flora isolated from soil, plant, and marine aquatic heavy-metal contaminated habitats. Furthermore, to assess the effect of soil microorganisms on Cd/Zn accumulation by *Helianthus annuus* L. and to examine the expression levels of ATPases (*HMA3* and *HMA4*) in root and shoot systems as representatives of metal homeostasis candidate genes.

## Results

### Isolation and phenotypic identification of bacteria

Fifty-two bacterial colonies were isolated on the surface of Nutrient agar (NA), Pseudomonas agar (PA), Azotobacter and Azospirillum media. These isolates were obtained from rhizosphere soil, root and marine sea water samples collected from four heavy-metal contaminated loci. Specifically, 32, 17 and 3 bacterial isolates were obtained from soil, root and marine sea water samples, respectively. The plants from which the rhizosphere soil or root system were examined for bacteria were identified as the following: a plant species of Fabaceae family, *Sonchus oleraceus, Anthemis* sp., *Mesembryanthemum crystallinum*, *Nicotiana glauca*, and *Mesembryanthemum crystallinum* for S1A, S1B, S2A, S2B, S3A and S3B spots, respectively. The bacterial cell shapes reported in the collected samples ranged from cocci, rod-shaped and pleomorphic.

### Biochemical identification using VITEK2 system

The VITEK2 system was used for biochemical identification of 22 bacterial cultures. *Pseudomonas aeruginosa, Pseudomonas fluorescens, Pseudomonas putida, Pseudomonas mendoci, Bacillus subtilis, Bacillus thuringiensis, Bacillus atrophaeus, Bacillus cereus, Bacillus mycoides, Aneurinibacillus aneurinilyticus, Achromobacter denitrificans, Staphylococcus warneri, Sphingomonas paucimobili* and *Stenotrophomonas maltophilia* were among the bacterial species identified using this approach. The identification percentage ranged from 85–99%. Overall, the *Pseudomonas* species was the most common in the samples collected, and it was isolated in every location analyzed. In terms of Gram's reaction, 13 isolates were Gram-negative and 9 isolates were Gram-positive, respectively (Table 1). Supplementary Tables 3, 4 and 5 present the comprehensive results of the biochemical characterization.

**Table 1:** Bacterial isolates source, medium, Gram's reaction, cell shape and VITEK 2 microbial identification and probability percentage

Isolate No	Laboratory code	Source of isolation	Isolation medium	Gram reaction	Cell Shape	VITEK identification*	VITEK probability %
1	1BRY	S1B Root	Pseudomonas Agar	Gram positive	Rod	*Bacillus thuringiensis*	85
3	1BRB	S1B Root	Pseudomonas Agar	Gram positive	Rod	*Aneurinibacillus aneurinilyticus*	90
19	2AS2	S2A Soil	Nutrient Agar	Gram positive	Rod	*Bacillus atrophaeus*	95
2	2BSO	S2B Soil	Nutrient Agar	Gram positive	Rod	*Bacillus subtilis*	96
14	1ASB	S1A Soil	Pseudomonas Agar	Gram negative	Rod	*Pseudomonas aeruginosa*	99
17	1ASG	S1A Soil	Pseudomonas Agar	Gram negative	Rod	*Pseudomonas aeruginosa*	99
13	2BR2	S2B Root	Nutrient Agar	Gram negative	Rod	*Pseudomonas aeruginosa*	99
11	3BRB	S3B Root	Pseudomonas Agar	Gram negative	Rod	*Pseudomonas aeruginosa*	99
15	3BRG	S3B Root	Pseudomonas Agar	Gram negative	Rod	*Pseudomonas aeruginosa*	99
5	1BSB	S1B Soil	Pseudomonas Agar	Gram negative	Rod	*Pseudomonas aeruginosa*	99
12	1BSG	S1B Soil	Pseudomonas Agar	Gram negative	Rod	*Pseudomonas aeruginosa*	99
16	2BRY	S2B Root	Pseudomonas Agar	Gram negative	Rod	*Pseudomonas fluorescens*	91
4	2ASO	S2A Soil	Nutrient Agar	Gram negative	Rod	*Achromobacter denitrificans*	99
10	2ASB	S2A Soil	Nutrient Agar	Gram positive	Cocci	*Staphylococcus warneri*	98
18	2BSY	S2B Soil	Nutrient Agar	Gram negative	Rod	*Sphingomonas paucimobili*	95
20	Marine isolate 1	Sea water	Pseudomonas Agar	Gram negative	Rod	*Pseudomonas putida*	99
21	Marine isolate 3	Sea water	Pseudomonas Agar	Gram negative	Rod	*Pseudomonas mendocina*	99
22	Marine isolate 4	Sea water	Pseudomonas Agar	Gram negative	Rod	*Stenotrophomonas maltophilia*	99
8	2AR	S2A Root	Nutrient Agar	Gram positive	Rod	*Bacillus cereus*	89
9	2AS1	S2A Soil	Nutrient Agar	Gram positive	Rod	*Bacillus subtilis*	96
7	2BR1	S2B Root	Nutrient Agar	Gram positive	Rod	*Bacillus mycoides*	88
6	2BSB	S2B Soil	Nutrient Agar	Gram positive	Rod	*Bacillus subtilis*	96

^*^VITEK2 microbial identification system version 07.01 (biomerieux, France)

### Evaluation of the plant growth promoting properties of the soil, root and marine bacterial isolates

**Fig. 1:**
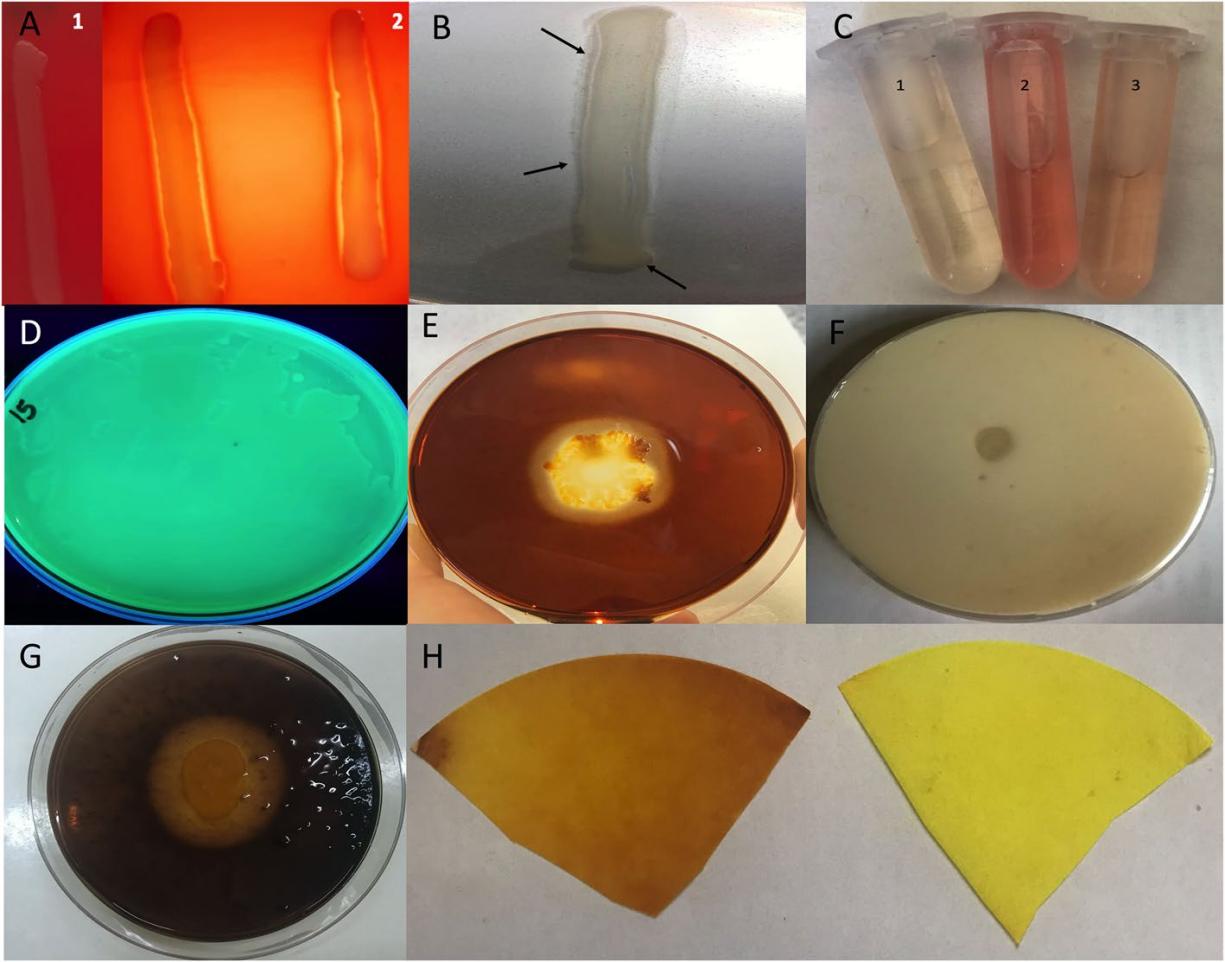
Examination of plant growth promoting traits for some bacterial isolates originally from rhizosphere soil, root system and sea water heavy metal contaminated loci (**A**) blood hemolysis test showing gamma-hemolytic (1) versus beta-hemolytic isolates (2). **B** Phosphate utilization referred to by black arrows on the surface of Pikovskaya’s Agar medium. **C** Red or faint red color formation upon the addition of Salkowski reagent for Indole-3-acetic acid (IAA) detection. **D** Fluorescent pigment formation on the surface of King’s B Agar medium by isolate number 15 (identified as *Pseudomonas aeruginosa*). **E, F** and **G** Detection of cellulose, casinase and chitinase enzymes production on the surface of carboxymethylcellulose (CMC), skim milk agar media and colloidal chitin containing-medium, respectively. **H** Hydrogen cyanide gas production (orange brown color) on filter paper versus control (yellow color)

Each bacterial isolate was assessed for a number of plant growth-promoting qualities. Four bacterial isolates were found to be beta-hemolytic or generate a complete blood hemolysis. As a result, these isolates (isolates number 6, 7, 8 and 9) were omitted from further analyses because they may represent a risk to the environment in future applications. In terms of thermostability of the tested isolates, only eight of them were able to grow at 50 °C, and only six isolates were able to survive at the higher temperature of 70 °C. At 70 °C, isolates 1, 3, 19, 2, 16, and 4 were able to withstand this high temperature (Table 2). Following that, each isolate's capacity to hydrolyze two polysaccharides, cellulose and chitin, or a phosphoprotein, such as casein, was tested. There were eight, nine, and fourteen isolates, respectively, able to hydrolyze cellulose, casein, and chitin, implying that chitinolytic bacteria were the dominant in these contaminated loci, regardless of the source, as this enzyme activity was found in rhizosphere, soil, root, and sea water isolates. Only three isolates, *Bacillus atrophaeus, Bacillus subtilis* and *Achromobacter denitrificans*, were able to hydrolyze the three tested substrates. Interestingly, seven *Pseudomonas aeruginosa* isolates identified in this study, namely isolates number 5, 11, 12, 13, 14, 15, and 17, were all yellow-green fluorescent pigment producers and were found to generate this pigment independent of the growth medium type. For instance, the pigments were detected on the surface of NA, PA, and King's B Agar media. Pyoverdine (a yellow-green fluorescent pigment) is known as the *Pseudomonas aeruginosa* most important siderophore [21]. The majority of the isolates tested positive for phosphate utilization and IAA synthesis, as 14 and 15 of the tested 18 isolates mentioned in Table 2 were able to consume phosphate and produce IAA, respectively. All *Pseudomonas* sp. isolates, both terrestrial and marine, were positive for both assays. Only *Bacillus subtilis* isolate number 2 was found to be positive in both tests. In terms of gas production, only 6 bacterial isolates were able to release HCN gas, which changed the color of filter paper impregnated with 2% sodium carbonate in 0.5 percent picric acid solution from yellow to orange brown. Two of the six HCN-producers were of marine origin, three were *Bacillus* genus members, and one was identified as *Sphingomonas paucimobili*. This gas could not be produced by any of the terrestrial *Pseudomonas* sp. isolates (Table 2). Figure 1 shows exemplified results for the investigated plant growth boosting qualities in the tested isolates.

**Table 2:** Evaluation of plant growth promoting properties of the selected rhizosphere soil, plant and marine isolates collected from heavy metal contaminated sites located in Alexandria, Egypt

Strain No	Laboratory code	VITEK identification	Blood Hemolysis	Thermostability at 50 ℃	Thermostability at 70 ℃	Cellulase	Casinase	Chitinase	Phosphate utilization	IAA production	Fluorescent pigment	HCN gas production
1	1BRY	*Bacillus thuringiensis*	Alpha-hemolysis	√^a^	√	√	x	√	√	x	x	√
3	1BRB	*Aneurinibacillus aneurinilyticus*	Alpha-hemolysis	√	√	x	x	x	x	√	x	x
19	2AS2	*Bacillus atrophaeus*	Alpha-hemolysis	√	√	√	√	√	x	√	x	√
2	2BSO	*Bacillus subtilis*	Alpha-hemolysis	√	√	√	√	√	√	√	x	√
14	1ASB	*Pseudomonas aeruginosa*	Alpha-hemolysis	x	x	x	√	√	√	√	√	x
17	1ASG	*Pseudomonas aeruginosa*	Alpha-hemolysis	x	x	√	x	√	√	√	√	x
13	2BR2	*Pseudomonas aeruginosa*	Alpha-hemolysis	x	x	x	√	√	√	√	√	x
11	3BRB	*Pseudomonas aeruginosa*	Gamma-hemolysis	x	x	x	x	√	√	√	√	x
15	3BRG	*Pseudomonas aeruginosa*	Alpha-hemolysis	√	x	x	√	√	√	√	√	x
5	1BSB	*Pseudomonas aeruginosa*	Alpha-hemolysis	x	x	x	√	√	√	√	√	x
12	1BSG	*Pseudomonas aeruginosa*	Alpha-hemolysis	x	x	x	x	√	√	√	√	x
16	2BRY	*Pseudomonas fluorescens*	Gamma-hemolysis	√	√	√	x	√	√	√	x	x
4	2ASO	*Achromobacter denitrificans*	Alpha-hemolysis	√	√	√	√	√	x	√	x	x
10	2ASB	*Staphylococcus warneri*	Gamma-hemolysis	√	x	x	x	x	√	x	x	x
18	2BSY	*Sphingomonas paucimobili*	Gamma-hemolysis	x	x	√	x	√	x	x	x	√
20	Marine isolate 1	*Pseudomonas putida*	Gamma-hemolysis	x	x	x	x	√	√	√	x	√
21	Marine isolate 3	*Pseudomonas mendoci*	Gamma-hemolysis	x	x	x	√	x	√	√	x	x
22	Marine isolate 4	*Stenotrophomonas maltophilia*	Gamma-hemolysis	x	x	√	√	x	√	√	x	√

^a^√ refers to positive result and X refers to negative result

### Molecular identification and phylogenetic analysis based on the 16S rRNA gene sequence.

**Fig. 2:**
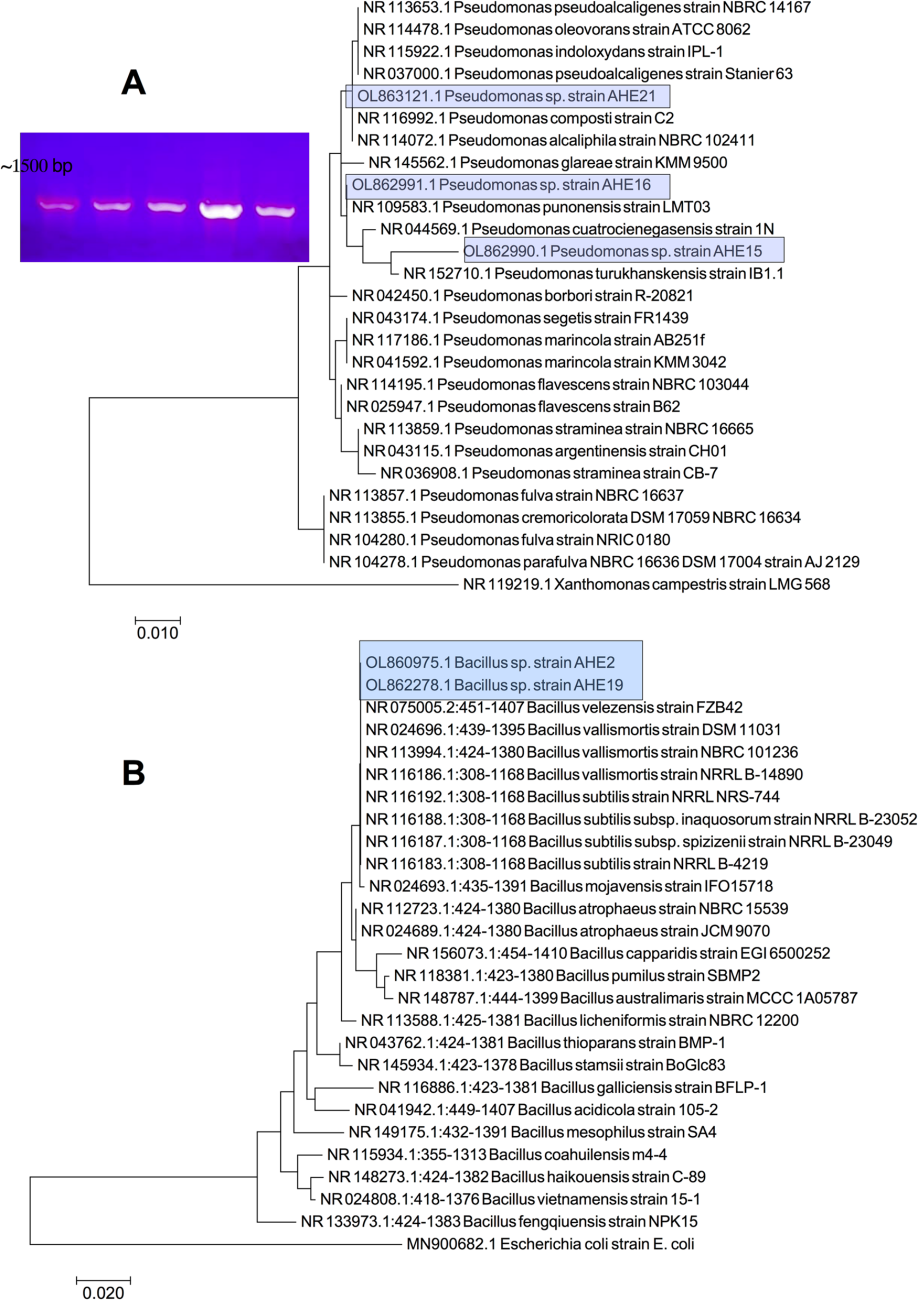
Phylogenetic analysis based on the 16S rRNA gene sequence for (**A**) *Pseudomonas* sp. isolates and (**B**) *Bacillus* sp. isolates after comparison with closely related homologs. The tree is based on the Maximum Likelihood method and bootstrapping (100 replicates). The evolutionary analyses were conducted in MEGA7. The gel image is for the amplified 16S rRNA gene of *Pseudomonas* sp. and *Bacillus* sp. isolates

Based on the detected plant growth promoting characteristics and diversity of the bacterial species, described in Table 2, five bacterial isolates were further identified on the molecular level. The obtained 16S rRNA sequences were deposited in the GenBank (NCBI) database under the following accession numbers: OL862990.1, OL862991.1, OL863121.1, OL860975.1 and OL862278.1 for *Pseudomonas* sp. strain AHE15, *Pseudomonas* sp. strain AHE16, *Pseudomonas* sp. strain AHE21, *Bacillus* sp. strain AHE2 and *Bacillus* sp. strain AHE19, respectively. The biochemical identification performed with the VITEK identification technology (Table 1) agreed with the molecular identification. As shown in Fig. 2A, phylogenetic analysis indicated that the three *Pseudomonas* sp. isolates included in this analysis are diverse and likely belong to distinct species of the genus *Pseudomonas*. The two *Bacillus* sp. isolates included in this analysis, on the other hand, were grouped in the same clade in the evolutionary tree by a 69 bootstrap value (Fig. 2B).

### Elemental content and pH of experimental soils

The total profile of HMs content in the different examined sites (shown in Table 3) indicated that the collected rhizosphere soils are polluted by more than one HM, notably Zn, Cu, Ni, and Cd, as determined by the atomic absorption technique. Because the soil at Site3 was significantly polluted with Zn and Cd, it was chosen for additional testing. The pH of the three tested sites ranged from 4.97 to 6.35.

**Table 3:** Element content and pH of the experimental soils collected

	**Soil element content (µg g**^**−1**^** dry soil)**											
	**0.01 M BaCl**_**2**_**-exchangeable**						**0.1 M HCL-extractable**					
**Site**	**S1**		**S2**		**S3**		**S1**		**S2**		**S3**	
	**Mean**	**SD**	**Mean**	**SD**	**Mean**	**SD**	**Mean**	**SD**	**Mean**	**SD**	**Mean**	**SD**
**Zn**	7.3794	1.9566	25.4300	4.9698	820.6000	12.7300	25.2330	2.3826	805.7000	17.1500	4173.0000	680.2400
**Cu**	0.0730	0.0082	0.6686	0.0605	0.8680	0.0900	4.8775	0.1701	33.2290	1.3650	32.7000	2.3400
**Ni**	1.4640	0.1276	0.1281	0.0197	0.9728	0.1618	4.9143	0.3637	11.9620	0.3395	4.2171	0.2638
**Cd**	0.0434	0.0066	0.0638	0.0125	0.5787	0.0257	0.3619	0.0087	0.3676	0.0264	8.4495	0.2686
**pH**	4.9760	0.2861	6.3520	0.2662	6.1220	0.1332						

### The effect of soil microorganisms on Cd/Zn accumulation by *Helianthus annuus* L.

*Helianthus annuus* L. or sunflower plants grown in untreated soil showed about 2.5 folds higher of cadmium accumulation in their shoots compared with plants grown on treated soil. Whereas their roots showed about 2 folds higher Cd compared with plant roots grown in treated soils. Moreover, *Helianthus* plants grown in untreated soil accumulated about 1.7 and 2.5 folds higher of zinc in their shoots and roots, respectively, compared with plants grown in treated soils (Table 4). These results imply that the microbiota is playing a crucial role in assessing the *Helianthus* plants to accumulate the Zn and Cd pollutants.

**Table 4:** Heavy metal concentrations in *Helianthus annus* shoots and roots

Element	Heavy metal concentration			
	**Shoot**		**Root**	
	**Untreated soil**	**Treated soil**	**Untreated soil**	**Treated soil**
**Cd (μg g-1 dry material)**	50.88 ± 3.2	20.89 ± 2.4	3.45 ± 0.32	1.83 ± 0.03
**Zn (mg g-1 dry material)**	11.33 ± 1.12	6.56 ± 1.8	5.57 ± 1.09	2.24 ± 0.14

### The expression levels of ATPases (*HMA3 *and *HMA4*) in root and shoot systems as representatives of metal homeostasis candidate genes

**Fig. 3:**
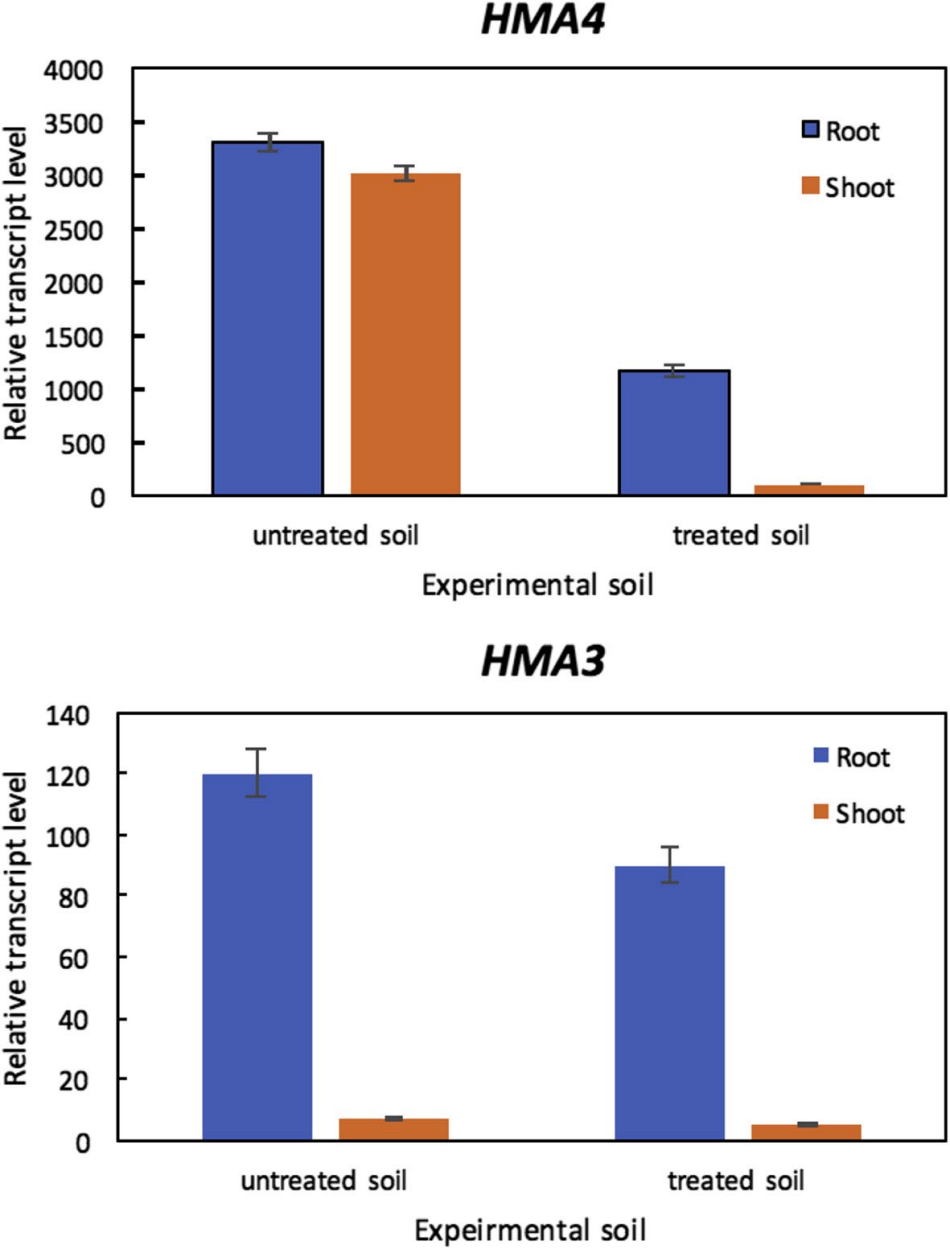
Real-time RT-PCR analysis of expression of *HMA3* and *HMA4* genes in *Helianthus annuus*. Transcript levels were assessed in 21 days old seedlings by real-time RT-PCR in roots and shoots of plants following 14 days of growth in untreated and treated experimental soils. Data shown are transcript levels relative to *EF1*⍺ from one experiment representative of two independent biological experiments. Tissues from three pots were pooled for each treatment. Values are mean ± s.e.m, (*n* = 3)

On the molecular level, the expression of *HMA3* and *HMA4* genes of *Helianthus* plants in roots and shoots were examined using Real-time RT-PCR. *Helianthus* plants demonstrated higher expression levels of both *HMA4* and *HMA3* genes in roots of *Helianthus* plants grown in untreated experimental soil. Whereas higher *HMA4* transcript levels were observed in shoots of *Helianthus* plants grown in untreated soil (Fig. 3).

## Discussion

The development of low-cost, environmentally-friendly and effective strategies to remediate and recover polluted lands and waters is a vital need. Phytoremediation is a critical part in achieving this goal. On the other hand, phytoremediation may be time-consuming, seasonal, and limited by high metal concentrations that are toxic to plants. As a result, improving plant growth as well as enhancing its hyper-accumulation capacity are critical requirements for efficient phytoremediation application under field conditions, where a variety of biotic and abiotic factors coexist. Therefore, in the current study the plant growth stimulating capabilities of microbial residents of rhizosphere soil, plant roots, and marine aquatic environments polluted by high heavy metal (HM) concentrations were investigated for possible use as bacterial biofertilizers. Furthermore, the role of these HMs-adapted microbiota in boosting the metal hyper-accumulation capacity of *Helianthus annuus* was evaluated.

*H. annuus* or sunflower was selected as it is well known to possess a significant propensity for hyper-accumulation [8]. Furthermore, *H. annuus* is a typical agricultural plant used for oil production and animal feed. Its adaptability to a wide range of soil conditions has facilitated its widespread cultivation [22]. Based on our experimental setup, sunflower plants cultivated in soils with HMs adapted bacteria were able to accumulate about 1.7–2.5-folds more Cd and Zn in their shoots and roots, than plants grown in soils without this HMs resistant microbiota (Table 4). Furthermore, both *HMA4* and *HMA3* genes achieved higher expression levels in the roots of *Helianthus* plants growing in untreated experimental soil. Whereas higher *HMA4* transcript levels were observed in shoots of *Helianthus* plants grown in untreated soil (Fig. 3). Previous comparative transcriptomic studies of metal hyperaccumulators and non-accumulators shoot and root, later confirmed by real-time qPCR, have about 30 metal homeostasis candidate genes which showed a higher expression in metal hyperaccumulators compared with non-accumulators [23]. Overexpression of *Noccaea caerulescens Heavy Metal ATPase 3* (*TcHMA3*) enhanced the tolerance to Zn and Cd. *NcHMA3* encodes a tonoplast transport protein specific for cadmium transport, which is responsible for sequestration of Cd into the leaf vacuoles [10]. *HMA4* (*Heavy Metal ATPase 4*) is a gene that encode a plasma membrane protein transition metals transporter. The encoded proteins enhance the partitioning of metals from the root into the shoot [10–12].

These enhanced HMs accumulation results in untreated soil-cultivated sunflower plants suggest that the Plant Growth Promoting Rhizobacteria (PGPR) plays a critical role in boosting this plant system's hyper-accumulation capability. Several PGPR have been associated with boosting the plant’s HMs hyper-accumulation capacity. Previous studies reported that they assist plants in absorbing, precipitating, oxidizing, and reducing HMs concentration in soil, reducing the metal stress on plants and enabling increased phytoextraction of metals by hyper-accumulators [24, 25]. On the other hand, the PGPR supports the plant growth through several PGP traits for example, phosphate utilization, IAA production, hydrolytic enzymes excretion, siderophores formation and HCN gas release [26].

As noticed among all tested isolates (Table 1,2), gram negative bacteria were the most dominate representing about 59.1% (13 bacterial isolates). However, the most abundant species among these isolates were *Pseudomonas* sp., followed by *Stenotrophomonas* sp., *Achromobacter denitrificans* and *Sphingomonas paucimobilis.* Most *Pseudomonas* sp. are identified as PGP bacteria as they play a significant role in plant growth promotion including biological plant pathogen control through enhancing systemic resistance [27]. *Pseudomonas fluorescens* is a model bacterium to assess plant–microbe interaction benefits, including plant growth promotion under biotic and abiotic stress [28]. Furthermore, this bacterium is known to have important traits in effectivity and what is called “bacterial fitness” including the ability of the strain to adhere to soil particles and to the rhizoplane, motility and prototrophy, in addition synthesis of antibiotics, siderophores, hydrogen cyanide and production of hydrolytic enzymes. Moreover, *Pseudomonas* retains PGP traits as phosphate solubilization, nitrogen fixation, phytohormone production, and iron chelation. Such multidimensional utility makes *Pseudomonas fluorescens* a bioagent of choice to be manipulated in the sustainability of agriculture field [29]. Besides, *Pseudomonas aeruginosa* are considered as a suitable candidate for cadmium remediation with high capacity up to 94.7% [30]. On the other hand, it was reported by [31] that *Achromobacter denitrificans* species showed an impressive ability to resist cadmium up to 100 mg/l. Additionally, *Sphingomonas paucimobilis* have a remarkable attribute to remediated Pb and Cd with high MIC value of 2000 ppm and 500 ppm for both Pb and Cd, respectively [32]. According to our results, about 40.9% (9 Strains) of the rhizo-isolates were gram positive in which *Bacillus* species were the omnipresent strains. *Bacillus* is a sophisticated ubiquitous bacterium not only in rhizosphere soil but also in all environments. Rhizospheric isolates of *Bacillus* sp. showed a significant ability to promote plant growth by various means, such as production of phytohormone precursor including indole-3-acetic acid production, phosphate solubilization, and siderophore production besides they could serve as biocontrol agents [33]. *Bacillus* sp. isolated from the cadmium hyper-accumulator *Solanum nigrum* L showed a specifically uptake of about 75.78%, 80.48%, 21.25% of Cd (II), Pb (II) and Cu (II), respectively, under the initial concentration of 10 mg/l [34]. Screening of microbial diversity in the rhizosphere of *Helianthus* plants grown in contaminated soil affect both the accumulation of HMs and hence the expression levels of markers genes involved in the plant metal homeostasis. PGPRs have an effective role in the immobilization of heavy metals, precipitation, complex formation and adsorption [35]. We hypothesized that the results might indicate the role of soil microbial communities in immobilization and complexation of metals. However, more evidence is required.

Interestingly, the three marine isolates isolated in this study possessed a PGP properties comparable to their terrestrial counterparts (Table 2, Fig. 1). They were identified using the VITEK2 system as being *Pseudomonas putida*, *Pseudomonas mendocina* and *Stenotrophomonas maltophilia*. These isolates are NaCl-tolerant, making them promising candidates for future uses not just in HM-contaminated land and water surfaces, but also in salty environments. Due to the climate change and desertification, there is a sharp increase in salty land spaces [13, 14]. Inoculating salty fields with halotolerant plant growth promoting bacteria has recently been highlighted to be an environmentally acceptable and sustainable method of overcoming salinity and enhancing crop growth and yield under high saline conditions [36].

## Conclusions

Overall, the isolated terrestrial and marine isolates possessed PGP qualities that qualify them as good candidates for bioremediation of multi-HMs contaminated habitats.The soil microbial communities have an effect on the accumulation of HMs by plants. Furthermore, they improved the *H. annuus* metal absorption from soil especially for Zn and Cd and hence altered the metal homeostasis genes involved in the uptake, translocation and sequestration of metals. The results suggest the use of PGPR and *Helianthus* plants as a promising sustainable tool for HMs phytoextraction and contaminated sites cleaning. Marine isolates may serve for future work to study the crosstalk between high soil salinity and heavy metal contamination environmental problems.

## Methods

### Samples collection and processing

Experimental rhizosphere soils and plants were collected at three natural sites hosting different plant populations in the West of Alexandria based on industrial activities in these sites: site1 (S1) (N 31.1206665, E 29.817127), site2 (S2) (N 31.065150, E 29.769752) and site3 (S3) (N 31.163115, E 29.929042) in early 2020. Soils and plants were collected at two different spots from each site and marked as S1A, S1B, S2A, S2B, S3A and S3B. For sea water samples, water was collected from three different spots from the same site located in Eastern Harbour of Alexandria, Egypt, an area reported previously to have HMs pollution by Cd, Cu and Sn [37]. Briefly, one gram/milliliter of soil, root and sea water samples were serially diluted and cultured using the pour plate technique on the surface of Nutrient agar (NA), Pseudomonas agar (PA), Azotobacter and Azospirillum media (HiMedia Laboratories, LLC). Unique colonies were sub-cultured after 2–3 rounds of purification for biochemical identification.

### Elemental content and pH of experimental soils

From each pool, a subsample of 100 g of soil was air-dried in the laboratory at room temperature for one week in a paper bag, then sieved through a 2-mm mesh analytical sieve. Soil pH, total, extractable and exchangeable metal concentrations were determined by atomic absorption.

### Extractable metals

Subsamples of 1 gm soil were mixed with 10 ml 0.1 M HCl in 15 ml round-bottom polypropylene screw-cap Greiner tubes, vortexed and then shaken for 30 min using an overhead shaker (150 rpm at RT) for 1 h. Samples were filtered through Whatman No. 1 filter paper and 1 mL 65% (w/w) HNO_3_ was added [2].

### Exchangeable metals

Subsamples of 1 g soil were mixed with 10 ml 0.01 M BaCl_2_ in a 15-ml conical bottom centrifuge tube (TPP, Trasadingen, Switzerland) and then shaken using an overhead shaker (150 rpm at RT) overnight. Extracts were filtered through Whatman No. 1 filter paper, followed by the addition of 1 ml 65% (w/w) HNO_3_. Element concentrations (Zn, Cu, Ni, Cd) were determined in technical triplicates by atomic absorption (Analytik Jena AG—contrAA 300—High-Resolution Continuum Source Atomic Absorption Spectrometer for Flame and Hydride) [2, 23, 38, 39]

### Soil pH analysis

Samples of 3 g soil were mixed with 7.5 ml of 0.01 M CaCl_2_ in a 15-ml conical bottom polypropylene screw-cap Greiner tube vortexed thoroughly and then shaken using an overhead shaker (150 rpm at RT) overnight. Samples were centrifuged (2,000 g at RT) and pH was measured in the supernatant using a pH meter [2].

### Phenotypic and biochemical characterization of bacterial isolates

Initial phenotypic characterization of the isolated 52 bacterial cultures was performed to select the morphologically unique bacterial colonies in each sample. Each isolate was Gram stained and microscopically examined (1000X oil immersion lens) for subsequent full biochemical characterization. Based on phenotypic examination, only 22 bacterial cultures were selected for biochemical analyses and preserved in 50% glycerol stocks at -20 °C, the VITEK2 (BioMérieux, France) system for bacterial identification was adopted.

### Evaluation of plant growth promoting traits

A set of experiments were conducted on selected bacterial isolates to validate the plant growth promotion properties. Specifically, each bacterial strain was tested for the following:

### Biosafety test

The type of blood hemolysis (alpha, beta, gamma) was checked for each isolate to guarantee the safety of the selected strains for future bioremediation applications. Beta-hemolytic isolates were excluded from the following tests.

### Protease, cellulase and chitinase enzymes activity

The ability to produce diverse enzymes was examined on the surface of different media. For protease enzyme activity, the formation of clear zone surrounding the growth of each bacterial isolate was examined on the surface of skim milk agar. The ability of the bacterium to produce cellulase and chitinase was checked on the surface of carboxymethylcellulose (CMC) agar and colloidal chitin-containing (10 g/l) agar media, respectively. The formation of clear zone upon the addition of Gram’s iodine solution next to the bacterial growth on the surface of the previously referred two media was considered a positive result [40].

### Phosphate solubilization and Indole-3-acetic acid production (IAA)

Each bacterial isolate was cultured on the surface of Pikovskaya’s Agar [41]. Visible clear holes around the bacterial colony indicated a phosphate utilizer. Production of Indole-3-acetic acid (IAA) was evaluated for each bacterial isolate using the protocol adopted from [42].

### Thermotolerance testing

Each bacterial isolate was tested for its thermotolerance to high temperature incubation conditions. Specifically, each isolate was cultured on nutrient broth and incubated for 48 h at 50 °C and 70 °C, separately followed by re-incubation under favorable conditions at 30–32 °C.

### Hydrogen cyanide production

The HCN gas production was detected following the protocol developed by [43]. Briefly, the bacterial cells were cultured on the surface of King’s B Agar medium supplemented with glycine 4.4 g/l, simultaneously a sterile filter paper was dipped in freshly prepared 2% sodium carbonate in 0.5% picric acid solution. Afterwards, the soaked filter paper was placed in the top of the plate. A parafilm sealed plates were then incubated for 4 days at 30 °C. The development of orange-brown color indicated the production of HCN [43].

### Genotypic characterization of selected isolates

#### Genomic DNA extraction and amplification of the 16S rRNA gene

The bacterial DNA was isolated from five selected isolates following the manufacturer’s instruction for gram negative and positive bacterial strains, of GeneJET™ Genomic DNA Purification Kit (Thermo Fisher Scientific, UK). The amplification of the 16S rRNA gene was carried out at 95 °C for 3 min, followed by 35 cycles of (95 °C for 30 s, 50 °C for 30 s, 72 °C for 90 s). A final extension step at 72 °C for 5 min. The obtained sequences were submitted to the GenBank (NCBI).

### Phylogenetic analysis

The obtained 16S rRNA gene sequences were divided into 2 groups: *Bacillus* and *Pseudomonas* species, followed by BLAST search of the sequences in each group against the NCBI database of bacterial 16S rRNA sequences. ClustalW was then applied to align these multiple sequences together. Finally, the Maximum Likelihood method and bootstrap of 100 replicates were used for evolutionary trees construction using the MEGA7 program [44, 45]. The 16S rRNA gene sequence of *Xanthomonas* and *E. coli* were used as phylogenetic tree outgroups in case of *Pseudomonas* sp. and *Bacillus* sp. tree, respectively. The selection of these outgroups was based on Saati-Santamaría et al., 2021 [46] and Wei Wang, 2009 [47], respectively.

### Evaluation of microbiota effect on hyper-accumulation capacity of *Helianthus annuus L*. (Microcosm setup)

As soil collected from site3 showed the highest levels of both Zn and Cd, it was selected for further experiments. For microcosm setup, regular quartz sand was cleaned with 6 M HCl for 48 h, neutralized to pH 5.5 by washing with water, dried at 25 °C, and baked at 180 °C for 4 h. The sand was mixed with soil at a ratio of 1:3 (wt/wt), in order to allow the plant to grow more easily on the soil. Half of the soil-sand mix was sterilized by autoclaving for 40 min at 121 °C (treated soil) the remaining half was not autoclaved (untreated soil).

For initial wetting of the dry soil to a water content of approximately 35% (wt/wt), a soil extract instead of pure water was used in order to avoid shifts in ionic strength and mobility of mineral-bound ions. The soil extract was prepared by mixing the soil with sterile water (ratio of 1:1.4) and incubated for 6 h after 10 min of sedimentation. The extract was sterilized by the same autoclaving method. One kilogram of sterile and nonsterile soil-sand mixes per plant microcosm was wetted with 50 ml of sterile soil extract and homogenized.

Seeds of *Helianthus annuus L*. were sterilized for 1 min in a 1–5% sodium hypochlorite (NaOCI) solution, then rinsing them once or twice with sterile water. Seeds were germinated in distilled water for 7 days. The 7-days old seedlings were transferred to the pre-set of microcosm. 50 ml of sterile distilled water was added to each pot. For each soil, three microcosms with five plants each were set up. Plants were grown for 14 days in the green house. The shoots and roots were ground in liquid nitrogen and stored at -80 ºC for further gene expression analysis. The homogenized material was dried at 60 °C for total elemental (Cd, Zn) analysis with ICP-OES.

### Elemental analysis of shoot

Subsamples of 15 to 20 mg dry, finely homogenized shoot material were digested in concentrated 65% (w/w) HNO_3_ at 190 °C and 1,600 W for 20 min in a MARSXpress microwave (CEM Microwave Technology, Matthews, NC, USA), and samples cooled to RT were diluted with ultrapure water (Milli-Q, Veolia PURELAB flex, Antony, France). Element analysis (Cd and Zn) was conducted by Inductively Coupled Plasma Optical Emission Spectrometry (ICP-OES) [2]. Composition of calibration standards for ICP-OES analysis of plant samples (Supplementary Table 1).

### Real-time RT-PCR

Total RNA was isolated from shoot and root tissues of plants using the Qiagen RNeasy plant RNA kit (Direct-zol) and treated with DNAse to eliminate any genomic DNA. All kits were used according to the manufacturer's instructions. Synthesis of cDNA was carried out with poly-dT oligonucleotide primers using the Ambion RETROscript^TM^kit. Primers for real-time RT-PCR were designed using PRIMER EXPRESS software (Supplementary Table 2). PCR reactions were performed in a 96-well plate with an AppliedBiosystems ABI Prism 7900 HT Sequence Detection System, using SYBR Green to monitor cDNA amplification. Equal amounts of cDNA, corresponding to approximately 1 ng of mRNA, were used in each PCR reaction. In addition, a PCR reaction contained 10 µl of qPCR mastermix (Eurogentec, LieÁge, Belgium), 0.6 ml of SYBRGreen and 5 pmol of forward and reverse primers (Eurogentec) in a total volume of 20 ml. The following standard thermal profile was used: 2 min at 50 ºC, 10 min at 95 ºC, followed by 40 cycles of 15 s at 95 ºC and 60 s at 60 ºC. Data were analyzed using 7900 HT sequence detection system software. Threshold cycle (C_T_) values were determined for each reaction at a threshold value of the normalized reporter R_n_ of 0.2 [12].

## Supplementary Information

**Additional file 1: Supplementary Table 1. **Composition of calibration standards for ICP-OES analysis of plant samples. **Supplementary Table 2. **Primers (**5’->3’**)used for 16S rRNA gene amplification and real-time PCR for plant genesexpression quantification. **Supplementary Table 3. **Biochemical test results of gram's negative bacterial species using the VITEK2 microbial identification system version 07.01 (biomerieux, france®). **Supplementary Table 4. **Biochemical test results of Gram's positive rod-shaped bacterial species using the VITEK2 microbial identification system version 07.01 (biomerieux, france®). **Supplementary Table 5. **Biochemical test results of Gram's positive spherical-shaped bacterial species using the VITEK2 microbial identification system version 07.01 (biomerieux, france®). **Supplementary Fig. 1. **Full gel electrophoresis image of 16S rRNA gene bands of *Pseudomonas* and *Bacillus* sp. isolates demonstrated in Fig. 2A.

## Data Availability

All data generated or analyzed during this study are included in this article and its supplementary information files.
