# Supplementary material for: Microbe- plant interaction as a sustainable tool for mopping up heavy metal contaminated sites
Source: BMC Microbiol. 2022 Jul 7;22:174. doi: 10.1186/s12866-022-02587-x (PMC9261045; doi:10.1186/s12866-022-02587-x)
Supplement: Supplementary file 1 — Additional file 1: Supplementary Table 1. Composition of calibration standards for ICP-OES analysis of plant samples. Supplementary Table 2. Primers (5’->3’)used for 16S rRNA gene amplification and real-time PCR for plant genesexpression quantification. Supplementary Table 3. Biochemical test results of gram's negative bacterial species using the VITEK2 microbial identification system version 07.01 (biomerieux, france®). Supplementary Table 4. Biochemical test results of Gram's positive rod-shaped bacterial species using the VITEK2 microbial identification system version 07.01 (biomerieux, france®). Supplementary Table 5. Biochemical test results of Gram's positive spherical-shaped bacterial species using the VITEK2 microbial identification system version 07.01 (biomerieux, france®). Supplementary Fig. 1. Full gel electrophoresis image of 16S rRNA gene bands of Pseudomonas and Bacillus sp. isolates demonstrated in Fig. 2A. [file 12866_2022_2587_MOESM1_ESM.pdf]

Supplementary Table 1. Composition of calibration standards for ICP-OES analysis of plant samples.

	Concentration (µg mL-1)					
**Element**	**Standard 1**	**Standard 2**	**Standard 3**	**Standard 4**	**Standard 5**	**wavelength (nm)**
**Cd**	0.1	0.25	0.6	2.5	10	228,8
**Zn**	0.1	0.5	2.2	10	50	606,2

Supplementary Table 2. Primers **(5'->3')** used for 16S rRNA gene amplification and real-time PCR for plant genes expression quantification.

Primer	Gene ID	Forward	Reverse
**HMA3**	HanXRQChr05g0142281	CTACACCATCTACGCTGTTTCT	GCCGTCGTTAAGGATTGCGATG
**HMA4**	HanXRQChr06g0179401	TTATACTATCTATGCCGTCTCC	GTACCATCGTTAAGAATTGCAATA
**27F & 1492R**	16S rRNA	AGAGTTTGATCCTGGCTCAG	GGTTACCTTGTTACGACTT

Supplementary Table 3. Biochemical test results of gram's negative bacterial species using the VITEK2 microbial identification system version 07.01 (biomerieux, france®).

ISOLATE NO./ Test	4	5	11	12	13	14	15	16	17	18	20	21	22
APPA	-	-	-	-	-	-	-	-	-	-	-	-	+
H2S	-	-	-	-	-	-	-	-	-	-	-	-	-
BGLU	-	-	-	-	-	-	-	-	-	-	-	-	+
ProA	-	+	+	+	+	+	+	+	+	-	+	+	+
SAC	-	-	-	-	-	-	-	-	-	+	-	-	-
ILATK	+	+	+	+	+	+	+	-	+	-	+	+	-
GlyA	-	-	-	-	-	-	-	-	-	-	-	-	-
O129R	-	+	+	+	+	+	+	-	+	-	+	-	-
ADO	-	-	-	-	-	-	-	-	-	-	-	-	-
BNAG	-	-	-	-	-	-	-	-	-	-	-	-	-
dMAL	-	-	-	-	-	-	-	-	-	-	-	+	-
LIP	-	-	-	-	-	-	-	-	+	-	-	+	+
dTAG	-	-	-	-	-	-	-	-	-	-	-	-	-
AGLU	-	-	-	-	-	-	-	-	-	+	+	-	-
ODC	-	-	-	-	-	-	-	-	-	-	-	-	-
GGAA	-	-	-	-	-	-	-	-	-	-	-	-	-
PyrA	+	-	-	-	-	-	-	-	-	+	-	-	-
AGLTp	+	-	-	-	-	-	-	-	-	-	-	-	-
dMAN	-	+	+	+	+	+	+	-	+	-	-	-	-
PLE	-	-	-	-	-	-	-	-	-	-	-	-	-
dTRE	-	-	-	-	-	-	-	+	-	-	-	-	-
SUCT	+	+	+	+	+	+	+	+	+	-	+	+	+
LDC	-	-	-	-	-	-	-	-	-	-	-	-	-

**IMLTa**	-	+	+	+	+	+	+	+	-	-	+	-	-
**IARL**	-	-	-	-	-	-	-	-	-	-	-	-	-
**dGLU**	-	+	+	+	+	+	+	+	+	+	+	+	-
**dMNE**	-	+	+	+	+	+	+	-	+	+	+	-	-
**TyrA**	-	+	+	+	+	+	+	+	+	+	+	+	-
**CIT**	+	+	+	+	+	+	+	+	+	-	+	+	+
**NAGA**	-	-	-	-	-	-	-	-	-	-	-	-	-
**IHISa**	-	-	-	-	-	-	-	-	-	-	+	-	-
**ELLM**	-	-	-	-	-	-	-	-	-	-	-	-	-
**dCEL**	-	-	-	-	-	-	-	-	-	-	-	-	-
**GGT**	-	+	+	+	+	+	+	+	+	-	+	-	+
**BXYL**	-	-	-	-	-	-	-	-	-	+	-	-	-
**URE**	-	-	-	-	-	-	-	-	-	-	-	-	-
**MNT**	-	+	+	+	+	+	+	-	+	-	+	+	-
**AGAL**	-	-	-	-	-	-	-	-	-	-	-	-	-
**CMT**	-	+	+	+	+	+	+	+	+	-	+	-	-
**ILATa**	-	+	+	+	+	+	+	+	-	-	+	-	-
**BGAL**	-	-	-	-	-	-	-	-	-	-	-	-	-
**OFF**	-	-	-	-	-	-	-	-	-	-	-	-	-
**BALap**	-	+	+	+	+	+	+	-	+	-	-	-	-
**dSOR**	-	-	-	-	-	-	-	-	-	-	-	-	-
**5KG**	-	-	-	-	-	-	-	-	-	-	-	-	-
**PHOS**	-	-	-	-	-	-	-	-	-	-	-	-	+
**BGUR**	-	-	-	-	-	-	-	-	-	-	-	-	-
**Probability %**	**99**	**99**	**99**	**99**	**99**	**99**	**99**	**91**	**99**	**95**	**99**	**99**	**99**

Supplementary Table 4. Biochemical test results of Gram's positive rod-shaped bacterial species using the VITEK2 microbial identification system version 07.01 (biomerieux, france®).

ISOLATE NO./ Test	2	6	19	8	9	7	3	1
BXYL	+	+	+	-	+	-	-	-
BGAL	+	+	-	+	+	-	-	-
APPA	-	-	-	-	-	-	+	-
ELLM	-	-	-	+	-	+	-	+
dMNE	-	-	+	-	-	+	-	-
BMAN	-	-	(-)	-	-	-	-	-
INU	+	+	-	-	+	-	-	-
OLD	-	-	-	-	-	-	-	-
LysA	-	-	-	-	-	-	+	-
PyrA	+	+	+	(+)	+	+	-	+
CDEX	-	-	-	+	-	-	-	-
MdX	-	-	-	-	-	-	-	-
dMLZ	-	-	-	-	-	-	-	+
PHC	-	-	-	+	-	-	-	+
dGLU	+	+	+	+	+	+	-	+
ESC	+	+	+	-	+	(+)	+	+
AspA	-	-	-	-	-	-	(+)	(-)
AGAL	+	+	+	-	+	-	+	(+)
dGAL	-	-	-	-	-	-	-	-
AMAN	-	-	-	-	-	-	-	+
NAG	-	-	-	+	-	+	-	+
PVATE	+	+	(-)	+	+	(-)	+	+
dRIB	+	+	(-)	+	+	+	-	+

**TTZ**	+	+	-	-	+	(+)	-	-
**LeuA**	+	+	(+)	-	+	(-)	+	+
**AlaA**	+	+	(-)	-	+	-	+	(+)
**GLYG**	+	+	-	-	+	-	-	+
**MTE**	+	+	-	+	+	-	-	+
**PLE**	+	+	+	-	+	-	-	(+)
**AGLU**	+	+	+	+	+	(-)	-	+
**PSCNa**	-	-	+	-	-	-	+	+
**POLYB_1**	+	+	+	+	+	+	+	+
**PheA**	+	+	+	+	+	-	+	+
**TyrA**	+	+	+	+	+	-	+	+
**INO**	+	+	-	-	+	-	-	-
**GlyA**	+	+	+	-	+	-	+	-
**IRHA**	-	-	-	-	-	-	-	-
**dTAG**	-	-	-	-	-	-	-	-
**NaCL 6.5%**	+	+	(-)	+	+	+	-	+
**ProA**	-	-	-	-	-	-	-	-
**BNAG**	+	+	-	+	(+)	+	-	+
**MdG**	+	+	+	-	+	-	-	+
**dMAN**	+	+	+	-	+	-	-	-
**BGLU**	+	+	+	-	+	-	-	+
**dTRE**	+	+	-	+	+	+	-	+
**KAN**	-	-	-	+	-	+	+	+
**Probability %**	**96**	**96**	**95**	**89**	**96**	**88**	**90**	**85**

Supplementary Table 5. Biochemical test results of Gram's positive spherical-shaped bacterial species using the VITEK2 microbial identification system version 07.01 (biomerieux, france®).

ISOLATE NO./ Test	10	Test	10
AMY	-	POLYB	-
PIPC	-	dGAL	-
dXYL	-	dRIP	-
ADH1	+	ILATK	+
BGAL	-	LAC	-
AGLU	-	NAG	-
APPA	-	dMAL	+
CDEX	-	BACL	-
AspA	-	NOVO	-
BGAR	-	NC6.5	+
AMAN	-	dMAN	-
PHOS	-	dMNE	-
LeuA	-	MBdG	-
ProA	-	PUL	-
BGURr	-	dRAF	-
AGAL	-	O129R	+
PyrA	+	SAL	-
BGUR	+	SAC	+
AlaA	-	DTRE	+
TyrA	-	ADH2s	-
dSOR	-	OPTO	+
URE	+	Probability %	98

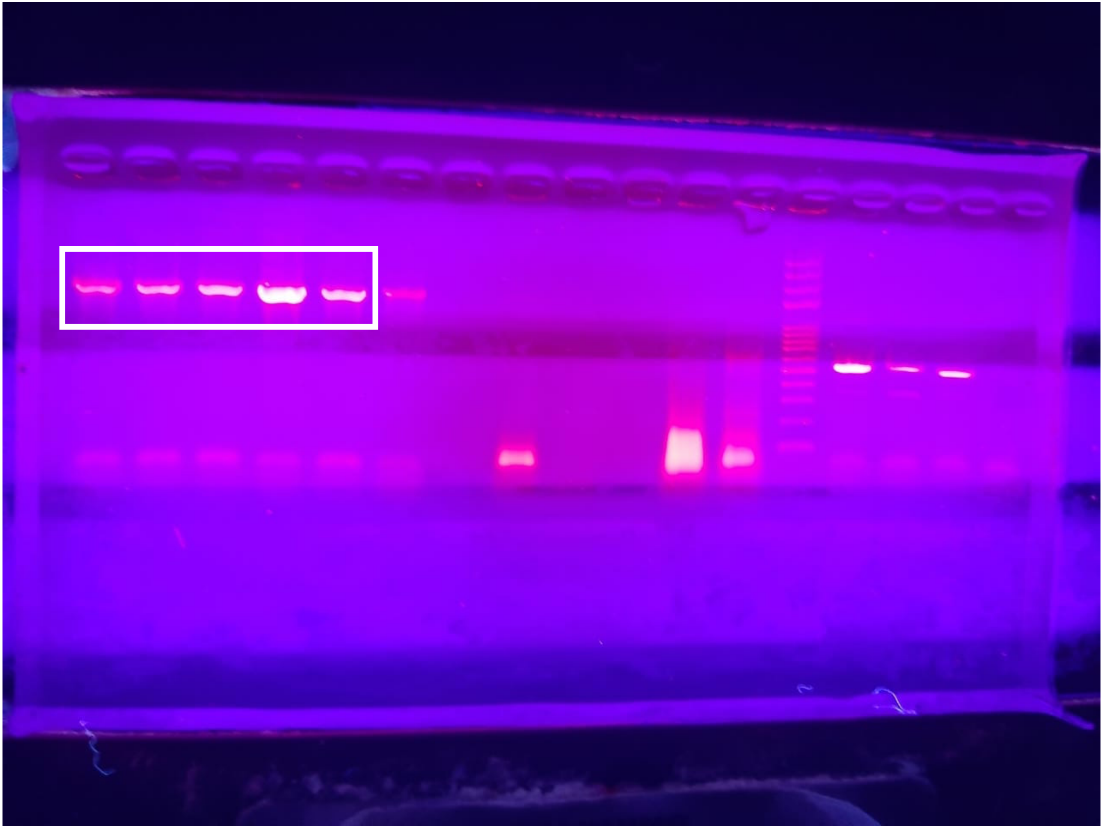

Supplementary Fig. 1. Full gel electrophoresis image of 16S rRNA gene bands of *Pseudomonas* and *Bacillus* sp. isolates demonstrated in Fig. 2A.
